# North Iowa Medical Society

**Published:** 1869-07-01

**Authors:** J. S. Green

**Affiliations:** Secretary


### *North Iowa Medical Society.*

The North Iowa Medical Society held its eleventh annual session at Monona, on Wednesday, the 3d of June. Meeting called in the office of Dr. Scott. Roll called—Minutes of last meeting read and approved.

Drs. Lewis L. Hazeltine, of McGregor, Herbert S. Hill, of Elkader and Dixon Alexander, of Fayette, were duly elected members of the Society.

Dr. Scott presented a little girl with a severe gun-shot wound of the hand, which he dressed, assisted by Green, Lewis and Hazeltine; the two middle fingers amputated through the metacarpal bones

Dr. Andros introduced a patient with chronic periostitis of the bones of the wrist. Most of the members gave their diagnosis and treatment in writing.

The Society then adjourned to meet at one o'clock, P. M., in the Masonic Hall. Meeting called at the appointed hour.

Dr. D. Mason, of Prairie du Chien, Wis., was elected Honorary Member.

A complaint was presented against Dr. J. Hicks, of Monona, which was referred to the Board of Censors, to draft resolutions to present to the meeting. Dr. Hicks was notified by the Secretary to appear and answer to the charges preferred against him. He would not appear.

The Society then passed the following resolutions :

WHEREAS, Dr. J. Hicks, of Monona, a member of this Society has been convicted of a *gross* violation of the code of the National Medical Association, said code being adopted by this Society, therefore, be it

*Resolved,* That he is hereby expelled from membership from this Society.

Dr. D. W. Chase, of Elkader, reported a case of progressive locomotor ataxy. Dr. Lewis a case of encephaloid disease of the forearm in a girl thirteen years of age, the diseased radius and ulna, with some of the encephaloid formation was exhibited. He also reported a case of the continued use of chloroform for eight hours. Dr. Scott one of six hours.

Dr. Green a case of pyœmia, with many large diffused abscesses which discharged, in all, over two quarts of pus; the patient recovered. Dr. Andros a case of abscess of the appendix vermiformis.

Dr. Earl a case of acute meningitis, accompanied with very unusual symptoms. Dr. N. H. Knowles exemplified Dr. Earle's case by one with similar symptoms, which resulted in death. A post mortem revealed also spinal apoplexy.

Dr. Earl one of organic disease of the brain, produced by functional disease of the uterus.

Dr. Scott read a very interesting and instructive paper upon various subjects pertaining to the medical profession.

Dr. Andros took his text from the modern bible: "Suffer not little children to come unto me," read a lengthy, true, and able discourse upon the common crime of criminal abortion; showed to what an alarming extent it is now carried on, the symptoms and treatment for the different stages; the danger attending it and the effect it had upon the patient, and upon the community at large ; showed by statistics that the American race is fast running out.

Many interesting cases were related, and then proceded to election, which resulted as follows:

D. W. Chase, of Elkader, President.

W. C. Earl, of Wawkon, Vice President.

J. S. Green, Postville, Secretary.

J. T. H. Scott, Monona, Treasurer.

Censors, Andros, Alexander and Lewis.

Delegates to the American Medical Association, F. Andros and N. H. Knowles.

Delegates to tho State Medical Society, Scott, Hill and Hazeltine.

Essayists, N. H. Knowles and J. S. Green.

The President was excused from reading his address for the want of time, as many of the members were compelled to leave on the train.

Meeting adjourned to meet at the City of McGregor, on the first Wednesday of June next. J. S. Green, *Secretary.*

---

### *Wines and Liquors for Medicinal Use.*

Attention is directed to the advertisement of C. H. Swain & Co., in the advertising pages of the Journal. We can confidently recommend this firm as reliable and prompt in their business relations, whilst their thorough acquaintance with *spiritual matters*, long experience and integrity entitle them to a goodly share of public patronage. If alcohol, in any of its various preparations, is to be used at all in professional practice, it is certainly of the highest importance to know exactly *what* is used, precisely as with any other medicament.

